# Identification of AQP3 and CD24 as biomarkers for carcinogenesis of gastric intestinal metaplasia

**DOI:** 10.18632/oncotarget.18817

**Published:** 2017-06-28

**Authors:** Haijian Zhao, Jianfei Wen, Xuqiang Dong, Ruji He, Cheng Gao, Weiming Zhang, Zhihong Zhang, Lizong Shen

**Affiliations:** ^1^ Division of Gastrointestinal Surgery, Department of General Surgery, First Affiliated Hospital, Nanjing Medical University, Nanjing 210029, Jiangsu, China; ^2^ Division of Gastrointestinal Surgery, Department of General Surgery, Affiliated Huai'an Hospital, Xuzhou Medical University, Huai'an 223002, Jiangsu, China; ^3^ Department of Pathology, First Affiliated Hospital, Nanjing Medical University, Nanjing 210029, Jiangsu, China

**Keywords:** gastric intestinal metaplasia, aquaporin 3, CD24, gastric cancer, pathology

## Abstract

Gastric intestinal metaplasia (GIM) is a precancerous gastric carcinoma (GC) lesion with pivotal roles in carcinogenesis. CD24, LGR5 and Ki67 are expressed in GIM; we previously demonstrated that aquaporin 3 (AQP3) is expressed in goblet cells and is positively correlated with GIM severity. However, the relationships of AQP3 with GIM classification and with other proteins, and their roles in the transition from GIM to gastric carcinoma (GC) remain unknown. Sixteen patients with intestinal-type GC were enrolled in this study. GIM was determined according to the updated Sydney system; GIM classification was determined via HID-AB staining, and AQP3, CD24, LGR5 and Ki67 expression were determined by immunohistochemistry. Type III GIM was more prevalent around the GC and displayed a positive association with GIM severity. CD24 was found in GIM, but LGR5 and Ki67 were found in tissues regardless of GIM. AQP3 expression showed significant correlation to type III GIM. CD24 expression was correlated with the marked GIM and incomplete GIM, while LGR5 expression decreased with GIM aggravation and did not have relationship with classification of GIM. However, Ki67 presented no association with GIM grade or classification. These observations identify AQP3 and CD24 as biomarkers for carcinogenesis of GIM, and may provide a precise strategy for screening at-risk candidates with GIM.

## INTRODUCTION

Gastric carcinoma (GC) remains one of the most common malignances and the third leading cause of cancer-related mortality worldwide [1]. However, the mechanism of GC carcinogenesis still needs to be elucidated. So far, it is well recognized that a multistep process is involved in the progression from normal gastric mucosa to intestinal-type GC, including chronic gastritis, chronic atrophic gastritis, intestinal metaplasia, dysplasia and invasive carcinoma, which was originally proposed by Correa [2, 3]. During this multistep procession, gastric intestinal metaplasia (GIM) is considered to be a precancerous lesion of GC and to play a pivotal role in GC tumorigenesis [4, 5]. Based on previous studies [6, 7], GIM is classified into three types: type I, type II and type III. According to whether the brush border is well-developed or not [6], GIM is classified as complete or incomplete. Type I GIM is complete GIM, and incomplete GIM includes type II and type III GIM. However, the relationship between GIM and GC remains controversial. Most studies agree that the incomplete GIM, especially type III GIM, has a higher GC risk than the complete GIM [8–10], whereas others believe that incomplete GIM has no association with the high prevalence of GC [11, 12].

GIM patients may eventually progress into intestinal-type GC [3, 13]; however, the incidence remains low [14, 15], and direct evidence of gastric carcinogenesis from GIM remains elusive. Some proteins, including the gastric cancer stem cell biomarker CD24 [16], leucine-rich-repeat-containing G-protein-coupled receptor 5 (LGR5) [16, 17] and the cell proliferation biomarker Ki67 [18], have been reported to be expressed in GIM tissues, and they are postulated to be involved in the progression from GIM to GC [15, 19, 20]. However, no further research has been performed to investigate the relationship of these proteins to GIM. Due to the association of GIM with GC, several guidelines have been recommended for the surveillance and screening of these precancerous conditions or lesions [21–23], especially GIM. However, there is no individualized strategy proposed for GIM surveillance in these guidelines, and it seems that no guideline for GIM surveillance has allowed for a patient-tailored approach [24].

Previously, we have demonstrated that aquaporin 3 (AQP3), a member of the aquaporin family, was expressed specifically in the membrane of goblet cells, and that AQP3 expression positively correlated with the severity of GIM [25], indicating that AQP3 may play an important role in gastric carcinogenesis from GIM. However, the relationship of AQP3 expression with the GIM classification, its cross-relationship with other proteins, i.e., CD24, LGR5 and Ki67, and their potential roles in gastric tumorigenesis from GIM remain unknown. In this study, we further refined the classification of GIM in the non-cancerous gastric mucosa adjacent to GC, as well as documenting the expression of AQP3, CD24, LGR5 and Ki67 in these tissues. We found that type III GIM was a more prevalent event in tissues around GC and correlated with marked GIM. We also showed that AQP3 and CD24 were expressed in non-cancerous mucosa tissues with GIM, while LGR5 and Ki67 were expressed in mucosa tissues regardless of the presence of GIM. AQP3 showed significant correlation to type III GIM, and CD24 exhibited remarkable association with the marked GIM and the incomplete GIM. In addition, LGR5 expression in GIM showed remarkable correlation with Ki67. These observations further establish the role of AQP3 in the gastric tumorigenesis, and suggest that CD24, rather than LGR5 and Ki67, may be involved in this progression. This study identifies AQP3 and CD24 as biomarkers for carcinogenesis of GIM, and improves our understanding of the mechanism of carcinogenesis from GIM to GC and may provide a precise strategy for screening at-risk candidates with GIM.

## RESULTS

### Type III GIM correlates with the severity of GIM

Previously, we reported that the incidence of GIM was 50% in 192 regions of the non-cancerous gastric mucosa tissues around the GC. We also showed that the incidence and severity of GIM was correlated with the distance from the GC, and GIM became more prevalent and more severe with increasing proximity to GC lesions. These data suggest an association between GIM and gastric carcinogenesis [25]. In this study, we identified the classification of GIM around GC. Among the 96 regions of mucosa tissues with GIM around GC, type III GIM occupied 52.1% (50/96), while type I GIM and type II GIM occupied 20.8% (20/96) and 27.1% (26/96), respectively, indicating that type III GIM was more prevalent in tissues adjacent to GC (χ^2^=23.625, *P*<0.001).

We then evaluated the correlation between the type of GIM with the distance from the GC, finding that there were no significant differences (Table 1; χ^2^=2.416, *P*=0.66). We further investigated the relationship between GIM type and GIM severity, and found that marked GIM was associated with type III GIM (Table 2; χ^2^=13.398, *P*=0.009). These results are consistent with previous studies [8], which indicated that incomplete GIM, especially type III, has a higher GC risk than complete GIM.

**Table 1:** Correlation between the type of GIM and the distance from GC

	Type of GIM			χ^2^	*P*
	I	II	III		
A	9	10	25	2.416	0.66
B	6	8	17		
C	5	8	8		

A, B and C represent ≤1 cm, 1–2 cm and >2 cm to the margin of the GC lesion respectively.

**Table 2:** Correlation between the severity of GIM and the classification of GIM

Type of GIM	Grade of GIM			χ^2^	*P*
	1	2	3		
I	7	7	6	13.398	0.009
II	2	11	13		
III	6	10	34		

### AQP3, CD24, LGR5 and Ki67 expression in GIM and their correlations with GIM grade and classification

As reported previously [25], AQP3 was expressed specifically in the membrane of goblet cells in GIM, and AQP3 expression positively correlated with the severity of GIM (*P*<0.001), but AQP3 expression had no significant correlation with the distance from GC lesions (*P*=0.376).

**Figure 1:**
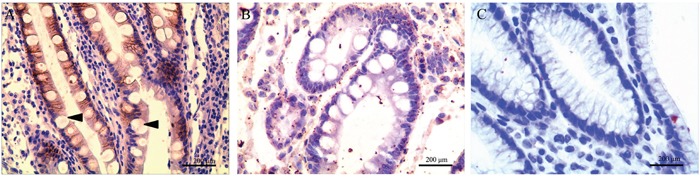
CD24 expression in gastric intestinal metaplasia (GIM) **(A)** Strong CD24 immunoreactivity in GIM; **(B)** negative CD24 expression in GIM; **(C)** CD24 was not found in tissues without GIM. CD24 was mainly located in the membrane and cytoplasm of columnar epithelial cells and was not expressed in goblet cells (arrow). Original magnification: 400×.

**Figure 2:**
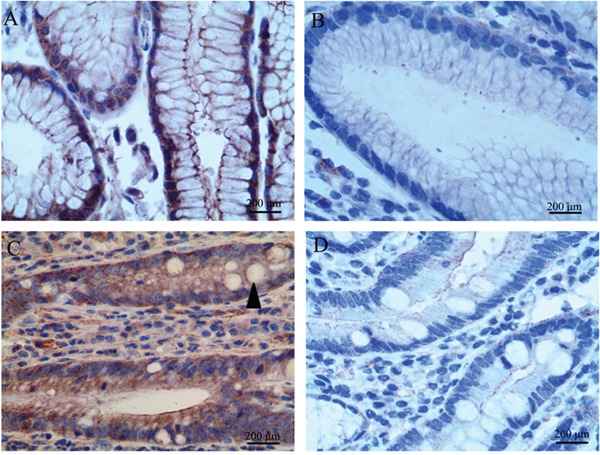
LGR5 expression in tissues adjacent to gastric carcinoma (GC) LGR5 expression was found in tissues regardless of the presence of GIM. **(A)** Positive LGR5 expression in tissues without GIM; **(B)** negative LGR5 in tissues without GIM; **(C)** strong LGR5 immunoreactivity in GIM; **(D)** negative LGR5 in GIM. LGR5 was mainly located in the membrane of columnar epithelial cells, and was not found in goblet cells (arrow). Original magnification: 400×.

**Figure 3:**
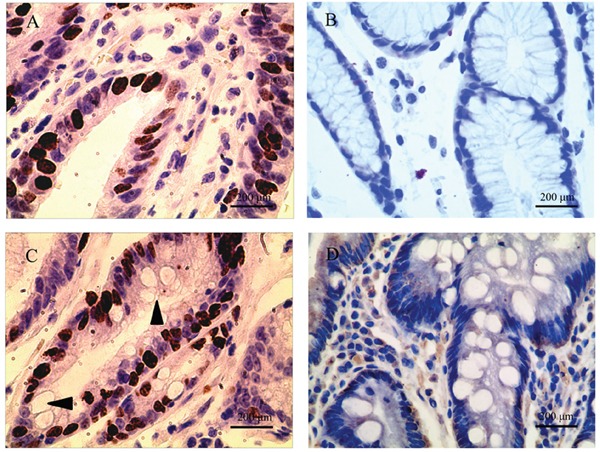
Ki67 expression in tissues adjacent to gastric carcinoma (GC) Ki67 expression was also found in tissues regardless of the presence of GIM. **(A)** Positive Ki67 expression in tissues without GIM; **(B)** negative Ki67 in tissues without GIM; **(C)** strong Ki67 immunoreactivity in GIM; **(D)** negative Ki67 in GIM. Ki67 was mainly located in the nucleus of columnar epithelial cells, and was not found in goblet cells (arrow). Original magnification: 400×.

In this study, we further investigated CD24, LGR5 and Ki67 expression in GIM. Unlike AQP3, these proteins were not specifically expressed in goblet cells. CD24 was expressed in the regions with GIM and was mainly located in the membrane and cytoplasm of columnar epithelial cells (Figure 1A), and its prevalence was 28.13% (27/96). CD24 was not found in goblet cells nor in tissues without GIM (Figure 1). LGR5 was found in tissues regardless of the presence of GIM, and the prevalence of LGR5 was 45.83% (44/96) in the regions without GIM (Figure 2A) and 20.83% (20/96) in the regions with GIM (Figure 2C). LGR5 was mainly located in the membrane of columnar epithelial cells and was not found in goblet cells. Like LGR5, Ki67 was also found in tissues despite the presence of GIM, and the prevalence of Ki67 was 40.62% (39/96) in the regions without GIM (Figure 3A) and 20.83% (20/96) in the regions with GIM (Figure 3C). Ki67 was mainly located in the nucleus of columnar epithelial cells and was not found in goblet cells. There was no significant correlation found between the expression of these proteins and distance from the GC (Table 3; r=-0.076, *P*=0.461 for CD24; r=-0.078, *P*=0.448 for LGR5; r=-0.142, *P*=0.168 for Ki67), which was similar to the results of AQP3 [25].

**Table 3:** Correlation of CD24, LGR5, Ki67 immunoreactivity with the distance from GC

	CD24				LGR5				Ki67			
	Positive	Negative	r	*P*	Positive	Negative	r	*P*	Positive	Negative	r	*P*
A	14	30	−0.076	0.461	11	33	−0.078	0.448	12	32	−0.142	0.168
B	8	23			5	26			5	26		
C	5	16			4	17			3	18		

A, B and C represent ≤1 cm, 1–2 cm and >2 cm to the margin of the GC lesion respectively.

We were more interested in the associations of AQP3, CD24, LGR5 and Ki67 with the grade and classification of GIM. In addition to the association with the severity of GIM, AQP3 showed significant correlation to the classification of GIM (Table 4; χ^2^=7.203, *P*=0.027) with the prevalence of 45%, 65.38% and 78% in type I, type II and type III GIM, respectively. As shown in Table 5, CD24 expression had a remarkable positive association with the severity of GIM (*P*<0.001), with this association being most prevalent in marked GIM (35.85%), while LGR5 also showed significant correlation with the grade of GIM (*P*=0.043) and LGR5 expression decreased with GIM aggravation. Ki67 was not found to correlate to the severity of GIM (*P*=0.126). Furthermore, there was also a significant association of CD24 (*P*=0.034) with the type of GIM, with the most prevalence in type II GIM (46.15%), but LGR5 and Ki67 exhibited no association with GIM type (Table 6; *P*=0.321 and *P*=0.204, respectively). Together, these data show that AQP3 and CD24, rather than LGR5 and Ki67, were associated with the marked GIM and the incomplete GIM.

**Table 4:** Relationship of AQP3 immunoreactivity with the subtypes of GIM

AQP3 immunoreactivity	Type of GIM			χ^2^	*P*
	I	II	III		
Positive	9	17	39	7.203	0.027
Negative	11	9	11		

**Table 5:** Correlation between CD24, LGR5, Ki67 expression and the grade of GIM

GIM grade	CD24				LGR5				Ki67			
	Positive	Negative	r	*P*	Positive	Negative	r	*P*	Positive	Negative	r	*P*
0	0	96	0.442	<0.001	44	96	−0.132	0.043	39	96	−0.101	0.126
1	2	13			3	12			3	12		
2	6	22			9	19			8	20		
3	19	34			8	45			9	44		

**Table 6:** Relationship of CD24, LGR5, Ki67 immunoreactivity with the classification of GIM

Type of GIM	CD24				LGR5				Ki67			
	Positive	Negative	χ^2^	*P*	Positive	Negative	χ^2^	*P*	Positive	Negative	χ^2^	*P*
I	6	14	6.751	0.034	4	16	2.273	0.321	7	13	3.182	0.204
II	12	14			8	18			5	21		
III	9	41			8	42			8	42		

### The cross-relationship of AQP3, CD24, LGR5 and Ki67 in GIM

We next evaluated the cross-relationship of AQP3, CD24, LGR5 and Ki67 in GIM. As shown in Table 7, AQP3 expression in GIM was not associated with CD24 (χ^2^=0.122, *P*=0.727), LGR5 (χ^2^=0.061, *P*=0.805) or Ki67 (χ^2^=0.061, *P*=0.805); CD24 expression in GIM exhibited no significant association with LGR5 (χ^2^=1.762, *P*=0.184) or Ki67 (χ^2^=3.559, *P*=0.059). However, LGR5 expression in GIM showed remarkable correlation with Ki67 (χ^2^=8.946, *P*=0.003).

**Table 7:** The cross-relationship of AQP3, CD24, LGR5 and Ki67 in GIM

AQP3	CD24				LGR5					Ki67				
	Positive	Negative	χ^2^	*P*	Positive		Negative	χ^2^	*P*	Positive		Negative	χ^2^	*P*
Positive	19	46	0.122	0.727	14		51	0.061	0.805	14		51	0.061	0.805
Negative	8	23			6		25			6		25		

CD24	Ki67				LGR5					
	Positive	Negative	χ^2^	*P*	Positive		Negative	χ^2^	*P*	
Positive	9	18	3.559	0.059	8		19	1.762	0.184	
Negative	11	58			12		57		

Ki67	LGR5		χ^2^	*P*
	Positive	Negative		
Positive	9	11	8.946	0.003	
Negative	11	65		

## DISCUSSION

Several epidemiological surveys have demonstrated that GIM is closely related to the development of GC [8–10]. Compared with patients with type I GIM and people with normal gastric mucosa, patients with type III GIM have a 4–11-fold increased risk of developing GC [26]. However, the pathological evidence is lacking with regard to the association between GIM and GC, and the underlying mechanisms remian to be elucidated. In this study, type III GIM was found to be a more common event than type I or type II GIM in the non-cancerous mucosa around GC, although there was no significant difference concerning the relationship of GIM type with the distance from GC lesions. Importantly, we also revealed that type III GIM displayed a remarkable positive association to the severity of GIM in the non-cancerous mucosa tissues around the cancer. These findings provide a pathological link between GIM and GC, especially type III GIM and GC.

However, the mechanism underlying the progression of GIM to GC has not been identified. CD24, LGR5, Ki67 and other proteins have been demonstrated to be expressed in GIM tissues [15, 19, 20], but their roles in this process need to be further investigated. Both CD24 and LGR5 are gastric cancer stem cell biomarkers expressed simultaneously in gastric cancer tissues [16], whereas Ki67 is involved in the formation of gastric adenocarcinoma [27]. This study investigated the expression of these proteins in GIM in addition to AQP3, and their significance in GIM was evaluated.

Aquaporins (AQPs) are a family of integral membrane proteins that transport water and, in some cases, water and glycerol (“aquaglyceroporins”) [28, 29]. We previously demonstrated that AQP3 is overexpressed in GC tissues and that its expression is associated with increased histological classification, lymph node metastasis and lymphovascular invasion [30, 31]. AQP3 upregulation promotes the proliferation and migration of GC cells via promoting epithelial-mesenchymal transition [32] and stem-like properties [33], suggesting that AQP3 is involved in the carcinogenesis and progression of GC. Interestingly, AQP3 was found to be expressed differentially in the membrane of goblet cells, and AQP3 immunoreactivity was identified more frequently in severe GIM areas [25]. In this study, AQP3 expression showed significant correlation to type III GIM. Collectively, these results indicated that AQP3 is expressed in GIM, and correlates with the most severe type of GIM, which supports the critical role of AQP3 in gastric inflammatory carcinoma transformation proposed in our previous study [25].

Our present study showed that CD24, LGR5 and Ki67 were also expressed in non-cancerous tissues around the GC. CD24 was expressed only in tissues with GIM, and its prevalence was rather low, while LGR5 and Ki67 were expressed in tissues regardless of the presence of GIM, and they were more common in tissues without GIM than that in tissues with GIM. Importantly, CD24 expression was found to be associated with type II and type III GIM. LGR5 expression decreased with GIM aggravation, but showed no correlation with GIM classification. Ki67 did not present any association with the grade and classification of GIM, and both LGR5 and Ki67 were not expressed in goblet cells. To our knowledge, AQP3 is the first and the only protein found to be expressed specifically in the membrane of goblet cells. These results indicate that AQP3 and CD24, rather than LGR5 and Ki67, may be involved in the carcinogenesis of GC from GIM.

CD24, an adhesive molecule and one of molecular biomarkers of cancer stem cells, is associated with cancer cell proliferation and migration [34, 35], and cells with CD24 expression may be the cancer-initiating cells that promote tumor migration and metastasis [36–38]. Wang and his colleagues reported that CD24 expression increased gradually in samples of normal gastric mucosa, non-atrophic chronic gastritis, chronic atrophic gastritis (CAG), CAG with intestinal metaplasia, dysplasia and GC [19]. Our results were consistent with this report and further support the potential role of CD24 in gastric inflammatory carcinoma transformation.

LGR5, identified as one of the biomarkers of gastric cancer stem cells, is associated with the carcinogenesis of gastric cancer [16, 17]. RNA *in situ* hybridization revealed the overexpression of LGR5 in intestinal metaplasia in the gastric antrum of mice [39]. Lineage tracing has confirmed cells with LGR5 expression to be the initiating cells of gastric adenomas in animal models [40–42]. However, such techniques are not suitable for studying human gastric cancer [43, 44]. Gastric stem cells with LGR5 expression were only found in the antrum of adult mice, where they drive self-renewal in the stomach and can be used to build long-lived gastric units *in vitro* [40, 45]. Ki67, a nuclear proliferation-associated antigen, is increased in many tumors and correlates with cell proliferation [18]. Studies have confirmed Ki67 expression in low grade adenoma, high grade adenoma and intestinal-type gastric adenocarcinoma [27], and its expression is increased in the transformation from GIM to GC [20]. Zheng et al reported that Ki67 expression was significantly higher in gastric carcinomas than in type I GIM, while no significant differences in Ki67 expression were observed among type II GIM, type III GIM and GC [46]. However, our study showed that Ki67 was expressed in adjacent mucosa tissues around the GC, but did not support their roles in carcinogenesis of GC from GIM, which remains to be further investigated.

Although AQP3 has been demonstrated to promote GC cell proliferation and the stem-like properties of human GC cells by activating the Wnt/GSK-3β/β-catenin signaling pathway [33], AQP3 expression in GIM was not found to have a relationship with CD24, LGR5 or Ki67 expression. We hypothesize that AQP3 may have different effects on non-cancerous cells from cancerous cells. In addition, the significant association of LGR5 and Ki67 in GIM needs to be investigated in the future.

At present, there is a paucity of recognized consensus on how to perform a follow-up for GIM due to the lack of convincing indicators for predicting the risk of transformation from GIM into GC, although *the role of endoscopy in the surveillance of premalignant conditions of the upper GI tract* (ASGE guideline) [21], and *management of precancerous conditions and lesions in the stomach (MAPS)* (European guideline) [23] have been recommended. The ASGE guideline does not recommend endoscopic surveillance for GIM uniformly; however, it agrees that patients at increased risk for GC due to ethnic background or family history might benefit from surveillance, and that endoscopic surveillance should incorporate a topographic mapping of the entire stomach. The MAPS recommends that endoscopic surveillance, every 3 years after diagnosis, should be offered to patients with extensive intestinal metaplasia, but it does not recommend endoscopic surveillance for patients with mild to moderate intestinal metaplasia restricted to the antrum. The Asia Pacific Working Group on Gastric Cancer [47] recommends the combined use of *H. pylori* serology, and serum gastrin-17 and pepsinogen concentrations [22], and the presence of histological intestinal metaplasia to screen gastric cancer [48]. However, these guidelines do not provide a patient-tailored approach for GIM surveillance. This study, as well as our previous report [25], provide the initial pathological evidence for the association of GIM severity and classification with gastric carcinogenesis, and show that AQP3 or CD24 expression correlates with the marked GIM and the incomplete GIM. We conceive that GIM severity and classification, as well as AQP3 or CD24 expression, should be introduced to surveillance programs for GIM. During surveillance, attention should be paid to the incomplete GIM and/or marked GIM, especially with AQP3 and/or CD24 expression. Thus, the high-risk patients will be identified and an individualized strategy can be implemented. As this is a preliminary study, a prospective and randomized clinical trial is needed to evaluate the feasibility and effectiveness of this approach.

## MATERIALS AND METHODS

### Human gastric tissue specimens

All human gastric tissue specimens of non-cancerous gastric mucosa tissues adjacent to GC presented in our previous study [25] were introduced in this study. These specimens came from 16 patients (median age: 62.25 ± 12.40 years; range: 44–86 years) diagnosed with intestinal-type gastric adenocarcinoma located in the lesser curve of the antrum between September and November 2014 at the Department of General Surgery, First Affiliated Hospital, Nanjing Medical University.

The gastric mucosal rolls in the four directions of 3-, 6-, 9- and 12-o'clock around the GC lesions, which corresponded to the posterior wall, pylorus, anterior wall and cardiac directions, respectively, were used in this study. We named this technique as “gastric mucosal sausage roll” [25]. The transverse sections of these rolls were obtained to evaluate GIM and immunoreactivity of AQP3, CD24, LGR5 and Ki67 by two experienced gastrointestinal pathologists that were blinded to the study. Each section was divided into three parts, A (≤1 cm), B (1–2 cm) and C (>2 cm), according to the distance to the margin of the GC lesion. The informed consent was obtained from all patients, and the protocol was approved by the Nanjing Medical University Institutional Review Board. This study was also in compliance with the Declaration of Helsinki.

### GIM grading and classification

Sections (5-μm-thick) were deparaffinized and stained with hematoxylin and eosin (HE). The presence of goblet cells indicated presence of GIM. According to the updated Sydney system [49], GIM were graded as absent, mild, moderate or marked (grades 0–3, respectively).

**Figure 4:**
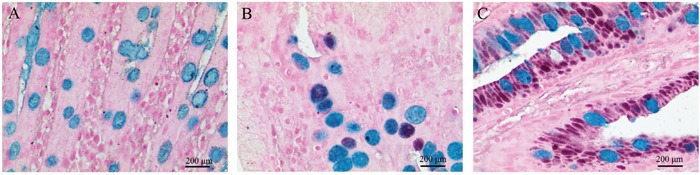
The classification of gastric intestinal metaplasia (GIM) with high iron diamine-alcian blue staining (HIDAB) **(A)** Type I GIM, sialomucin in goblet cells stained blue by HID-AB; **(B)** type II GIM, sialomucins and occasionally sulfomucins (black by HID-AB stain), or a mixture of these two mucins in goblet cells (stained brown/purple by HID-AB stain); **(C)** type III GIM, sulfomucins secreted in columnar intermediate cells, and sialomucins and/or sulfomucins secreted in goblet cells. Original magnification: 400×.

High iron diamine-alcian blue (HID-AB) staining was performed for GIM classification. Type I GIM presented sialomucin in goblet cells stained blue by HID-AB (Figure 4A). Type II GIM showed sialomucins and occasionally sulfomucins (black by HID-AB stain), or a mixture of these two mucins in goblet cells (stained brown/purple by HID-AB stain) (Figure 4B). Type III GIM presented sulfomucins secreted in columnar cells, and sialomucins and/or sulfomucins secreted in goblet cells (Figure 4C).

### Immunohistochemical assay for AQP3, CD24, LGR5 and Ki67 expression

AQP3, CD24, LGR5 and Ki67 expression in goblet cells was determined by immunohistochemistry (IHC), as described previously [25, 50]. The polyclonal rabbit anti-AQP3 antibody was obtained from Santa Cruz Biotechnology (Santa Cruz, CA, USA). The monoclonal mouse anti-CD24 and polyclonal rabbit anti-LGR5 antibodies were obtained from Abcam Biotechnology (Cambridge, UK) and the monoclonal rabbit anti-Ki67 antibody was from Fuzhou Maixin Biotechnology (Fuzhou, China). Two pathologists scored protein expression as the percentage of positive cells (scale 0%–100%) with a staining intensity from 0–3+. Positive IHC expression was defined as >25% staining with an intensity of 2–3+.

### Statistical analysis

All statistical analyses were performed with SPSS version 17.0 (SPSS Inc., Chicago, IL, USA). The association between various clinicopathological parameters was examined via Pearson's chi-square test and Spearman's rank correlation. *P*<0.05 was considered statistically significant.
